# A case of jejunal GIST revealed by hematemesis: Unusual situation

**DOI:** 10.1016/j.ijscr.2022.107146

**Published:** 2022-05-03

**Authors:** Behzad Azimi, Mohammad Amin Shahrbaf, Majid Iranshahi, Fatemeh Parsaeian

**Affiliations:** a Department of Vascular and Endovascular Surgery, Imam Hossein Hospital, Shahid Beheshti University of Medical Sciences, Tehran, Iran; b Faculty of Medicine, Shahid Beheshti University of Medical Sciences, Tehran, Iran; c Department of Internal Medicine, Imam Hossein Hospital, Shahid Beheshti University of Medical Sciences, Tehran, Iran

**Keywords:** Gastrointestinal stromal tumor, Jejunal tumor, Gastrointestinal malignancies, Upper gastrointestinal bleeding

## Abstract

**Introduction:**

Gastrointestinal stromal tumors (GISTs) are rare tumors of the gastrointestinal (GI) tract, which can occur in majorly stomach, and rarely in the small intestine, rectum, and large intestine. We report a jejunal GIST presented with massive hematemesis in the current study.

**Case presentation:**

A 39-years-old male, without any underlying history, was presented to our center with the complaint of massive hematemesis. Given the unstable hemodynamics of the patient, an upper GI endoscopy was requested, associated with a large amount of blood in the duodenal bulb without any sign of bleeding. The patient was sent to the operation room, and after laparotomy, a mass was seen in the jejunum, revealed as GIST after pathological study.

**Discussion:**

Small-intestine-related etiologies are rare conditions related to upper GI bleeding (UGIB). Jejunal GIST usually manifests as asymptomatic subepithelial mass and is associated with abdominal discomfort or GI bleeding. Sudden unset bleeding is a rare manifestation of jejunal GIST, but it can be associated with the emergency outcome and may need emergency intervention.

**Conclusion:**

UGIB can occur in jejunal GIST, which should be considered in the management of UGIB.

## Highlights

• Gastrointestinal stromal tumors (GISTs) are rare tumors of the gastrointestinal (GI) tract.
• GISTs are rarely found in the small intestine, compatible with abdominal discomfort.
• Jejunal GISTs usually manifest as intestinal obstruction and abdominal pain.
• Massive hematemesis is rare in jejunal GISTs and should be consider in the management of upper GI bleeding.

## 1 Introduction

Gastrointestinal stromal tumors (GISTs) are the most common type of gastrointestinal (GI) tract mesenchymal tumors, which can occur in all anatomical sites of the GI tract [1]. These tumors are usually located in the stomach, followed by the small intestine, rectum, and large intestine [2]. GISTs can be assumed as benign lesions without metastasis or associated with metastasis and have a broad spectrum of presentation from abdominal discomfort to severe conditions like peritonitis or shock [3]. This study reports a rare presentation of jejunal GIST with massive hematemesis identified by surgery, based on the Updating Consensus Surgical Case Report (SCARE) Guidelines [4].

## 2 Case presentation

### 2.1 Patient information

A 39-years-old male was presented to the emergency department with the chief complaint of massive hematemesis without any history of a similar condition. The hematemesis began a few hours before his presentation to our emergency department, and the patient was in the shock status (hypotension, tachycardia, and paleness) at the presentation. The patient had no past medical history regarding GI ulcers, no history of related medications including anticoagulants and nonsteroidal anti-inflammatory drugs (NSAIDs), no history of alcohol intake, no positive history of chronic liver diseases, or any history of previous GI bleeding. The history of familial or genetic predisposing was also negative in the patient.

### 2.2 Clinical findings

The patient's systolic blood pressure was 85 mm Hg, and the diastolic blood pressure was 65 mm Hg. In addition, the patient's heart rate was 120 beats/min, and the patient was in shock status. Considering the unstable hemodynamics of the patient, massive hydration with two liters of normal saline was started. Due to a low hemoglobin level, which was 6.5 g/dl, blood transfusion was started for the patient with two units of iso-group and iso-Rh packed cell and two units of fresh frozen plasma (FFP). Given the emergency status of the patient and lack of proper equipment such as dynamic CT angiography, which is indicated in this situation, the patient was sent to upper GI endoscopy for further investigation after adequate resuscitation. Other lab data were in the normal range.

In the upper GI endoscopy, conducted by the gastroenterologist (the third author), a large amount of fresh blood was observed in the stomach and duodenal bulb without any sign of active bleeding in these sites. It was revealed that the possible source of bleeding might be related to the distal of the duodenal bulb, and considering not finding the exact site of GI bleeding and the unstable condition of the patient, an emergency surgical consultant was requested for the patient and considering the unknown site of the massive bleeding related to GI tract, resulted in shock for the patient, the patient was taken to the operating room immediately for further exploration.

### 2.3 Therapeutic intervention

**Fig. 1:**
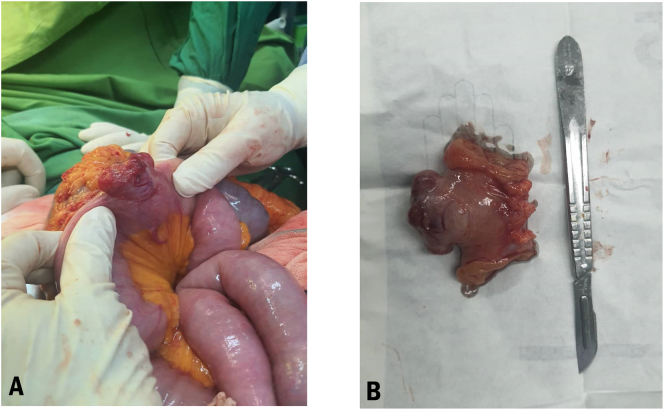
A: the jejunal mass which observed 30 cm distal to the Treitz ligament. As seen by the arrow, seromuscular artery is seen which is attributed to a possible GI tumor with AVM. B: The pathological specimen of the GI mass with the extent of 6 × 4 × 3.5 cm.

The first author conducted the principal procedure. After midline laparotomy and based on the endoscopy report, a 3 cm longitudinal duodenotomy was performed to explore the duodenal bulb as the primary site of the bleeding. There were no sites for active bleeding, and the duodenal mucosa was intact, although vast amounts of fresh blood were seen distal to the incision. Thus, we first explored the proximal site of the primary incision by conducting a 5 cm gastrotomy along grater curvature, which was accompanied by suction of a vast amount of dark blood without any apparent lesions. Furthermore, we explored the distal of the Treitz ligament and found a mass in the jejunum, about 30 cm from the ligament of Treitz. The mass seemed to be an arteriovenous malformation (AVM) or a GIST containing an AVM due to blood turbulence under the seromuscular layer (Fig. 1). The mass was removed by segmental resection, and the remaining parts anastomosed by an end-to-end anastomosis. In addition, the gastroduodenal incision was repaired with two layered of omental patch to prevent from possible fistula. A specimen was sent to the pathology for a definite diagnosis.

### 2.4 Pathological evaluation

**Fig. 2:**
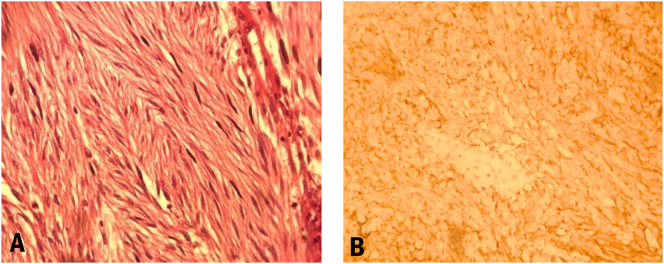
Microscopic pathological view of the tumor. A: Spindle shaped cell with Ovid nuclei. B: The positivity for DOG-1 in the IHC study.

The surgical specimen had a 6 × 4 × 3.5 cm extent with a gray, lobulated surface compatible with a GIST. In the microscopic evaluation, bland-looking spindle-shaped cells with Ovid-shaped nuclei and mild atypia were seen, suggesting a low-grade GIST. Moreover, immunohistochemical (IHC) studies showed the positivity of CD117 and discovered on GIST-1 (DOG-1) antigens accompanied by the negativity in Desmin and smooth muscle actin (SMA). The pathological view of the tumor is presented in Fig. 2.

### 2.5 Follow-up and outcomes

The postoperative period was without complications and after starting the oral feeding on the second day of the postoperative period, the patient was discharged in good clinical condition. Spiral lung CT and abdominopelvic MRI were conducted to assess the possible metastasis, which were normal. Imatinib was administered with the dose of 200 mg BD for three years, to subside microscopical metastasis. In the first year of follow-up, the patient tolerated the treatment well with no side effects.

## 3 Discussion

This study presents a case of jejunal GIST, which manifested as massive hematemesis. We managed it by surgical resection and started adjuvant therapy by Imatinib for three years. Our case was presented as sudden hematemesis; however, previous cases of jejunal GISTs usually present as intestinal obstruction [5], abdominal pain [6], or peritonitis [7], and hematemesis is usually seen in gastric GISTs [8], [9]. Hematemesis is a rare manifestation of jejunal GIST, which is valuable to mention to the best of our knowledge.

Upper GI bleeding (UGIB) is one of the life-threatening emergency conditions in general medicine, which manifests as acute hematemesis or melena [10]. The prevalence of this condition is sixfold higher than lower GI bleeding (LGIB), and it was more prevalent in males than females [11]. UGIB can be caused by several etiologies, including gastric or duodenal ulcers, esophageal or gastric varices, angiodysplasia, and malignancies [12]. Small-intestine related etiologies contain almost 5% of the UGIB etiologies and include etiologies such as Meckel's diverticulum, inflammatory bowel disease, celiac disease, vascular anomalies, and medication-related ulcers [13]. However, some rare conditions, including GIST, can cause UGIB [14].

GISTs are mesenchymal neoplasm of the GI tract, originate from the interstitial cells of Cajal, and include less than 0.2% of all GI tumors and only 0.04% of small intestine tumors [15]. These tumors can be found in any site of the GI tract; however, they are commonly found in the stomach (almost 50%) and jejunum (almost 30%) [16]. GISTs commonly manifest as asymptomatic subepithelial mass, which was discovered incidentally during endoscopy or surgery; however, some tumors are associated with abdominal pain, GI bleeding, bowel perforation, or GI obstruction [17]. The GIST presentation as GI bleeding was reported in almost 25% of jejunal GISTs [18]; however, the present case was asymptomatic, with no prior history of abdominal discomfort or GI bleeding, and manifested by sudden onset of massive hematemesis, which assumes as a rare manifestation of jejunal GISTs.

GISTs identified microscopically as spindle, epithelioid or mixed types of tumors by tyrosine kinase receptors over expressions [19]. However, it is hard to differentiate these lesions from other subepithelial tumors by microscopic evaluation, and an immunohistochemistry (IHC) study is necessary for differentiation [20]. These tumors can differentiate from other subepithelial lesions, including leiomyoma, leiomyosarcoma, GI cysts, and lipoma; through IHC by the positiveness of CD117 and KIT negativeness of Desmin and S100 [21]. The present case represents bland-looking spindle-shaped cells with the positiveness of CD117, which confirm the diagnosis of GIST.

Several treatment modalities are used for treating GISTs, including surgical resection, targeted drug therapy, chemotherapy, and radiation therapy; however, surgical resection is preferred due to undesirable responses to other modalities [22]. Endoscopic resection is not suggested due to the possible damage to the tumor's capsule, which increases the risk of recurrence [23]; however, complete surgical resection in primary GIST is associated with a 5-year survival rate of 30–65% [24]. We conducted a complete surgical resection in this case and started our chemotherapy program after the surgery for possible microscopical metastasis.

GISTs prognosis depends on tumor size, anatomical location, imaging features, metastasis, and tumor rupture [25]. Most GISTs may develop metastasis in the first year of follow-up; however, metastasis can occur up to 28 years after the diagnosis [26]; therefore, adjuvant therapy is necessary for GISTs. Imatinib, a tyrosine kinase receptor inhibitor, is usually used to prevent tumor recurrence after the surgery, in huge unresectable cases, or metastatic disease [27]. We used this drug for three years to prevent possible metastasis in our case. As previously regarded, it is better to administrate it twice daily, compared to once daily, or in comparison to administrating Doxorubicin to prevent metastasis in jejunal GIST [28].

In general, sudden bleeding and peritonitis can be a complication of jejunal GIST, which has been addressed in previous cases [29], [30]. However, previous cases majorly presented with lower GIB (LGIB). In the study of Mahmoud et al., jejunal GIST was presented with life-threatening melaena and hematochezia, which was different from our study [31]. In another study by Mohamed et al., massive LGIB, which required urgent surgical intervention, was seen in a 58-year-old female [32]. The current case presented with sudden UGIB, which is the rarity of our case. Mandala et al. reported a similar presentation in a patient with type 1 neurofibromatosis with concurrent melena and hematochezia [33]. In a recent publication by Liu et al., a novel case of jejunal GIST was presented in a young male with UGIB, with a chronic manifestation, which was different from our case [34].

### 3.1 Patient perspective

The patient was admitted to our center with volatile vital conditions. Despite the limited facilities, the patient was discharged with appropriate vital conditions. In addition, based on the available facilities, appropriate diagnosis and treatment were provided for the patient. At the first-year follow-up visit, the patient was satisfied with his treatment.

## 4 Conclusion

Massive GIB is one of the rare presentations of these tumors as the GIB that occur in GIST is chronic and diagnosed by anemia. Small intestine GISTs rarely occur, and a jejunal GIST that presents with massive GIB is unusual.

## Informed consent and ethical consideration

Written informed consent was obtained from the patient to publish this case report and accompanying images. A copy of the written consent is available for review by the journal's Editor-in-Chief.

## Provenance and peer review

Not commissioned, externally peer-reviewed.

## Ethical approval

This study was conducted under the consideration of Shahid Beheshti University of Medical Sciences.

## Funding

There is no source of funding for this study.

## Guarantor

Behzad Azimi, MD; Assistant Professor of Vascular Surgery.

## Research registration number

Not applicable.

## CRediT authorship contribution statement

B.A designed the study and conducted the surgical procedure, M.A.S drafted the manuscript, M.I conducted the gastrointestinal procedures, helped with the drafting of the manuscript, and F·P edited the manuscript.

## Declaration of competing interest

The authors declare no conflict of interests.
